# I win it’s fair, you win it’s not. Selective heeding of merit in ambiguous settings

**DOI:** 10.1371/journal.pone.0279865

**Published:** 2023-01-06

**Authors:** Serhiy Kandul, Olexandr Nikolaychuk

**Affiliations:** 1 Institute for Biomedical Ethics and Medical History, University of Zurich, Zurich, Switzerland; 2 Institute of Economic Research, University of Neuchâtel, Neuchâte, Switzerland; 3 Faculty of Economics and Business Administration, Friedrich Schiller University Jena, Jena, Germany; Universität Heidelberg, GERMANY

## Abstract

One’s willingness to accept an outcome or even to correct it depends on whether the underlying procedure is deemed legitimate. We examine a modified version of the dictator game, where dictatorship is assigned by a fair procedure that is linked to the participant actions but in effect is completely random, to illustrate that this belief is not independent of the outcome and is self-serving in its nature. We also discuss the perceptions of fairness and merit as potential drivers of the observed behavioral phenomenon.

## Introduction

There is a plethora of allocation procedures governing our everyday interactions that are conventional or in effect arbitrary in their nature. They serve as practical solutions in situations where merit, which typically underpins preferences over redistribution [1–3], is hard to define or when it is problematic to differentiate factors within individual control from those beyond it [4–8].

We posit that in the absence of clear causal links between actions and outcomes, subjective assessments of allocation procedures are inherently self-serving in that people are willing to grant merit to those actions (more generally, procedures) that serve their personal interests but are not willing to do so otherwise. As a result of such *selective heeding*, the haves and have-nots are likely to hold opposing views on the same outcome (and consequently, on the underlying procedure), yet if the positions were reversed, so would be the views.

To test this hypothesis, we construct a series of decision situations where pairs of human participants perform equally effortful actions that lead to a power imbalance between the two but that do not affect it in the causal sense. In contrast to the literature on purely random procedures, where the participants are not actually involved in the allocation of outcomes [9–11], we thus render merit attribution deliberately ambiguous. The empowered participant is then free to assign (relative) merit to their own action, which is elicited through their redistribution preferences.

Following [12, 13], we consider a procedure to be fair if a priori all participants have an equal chance to obtain the favorable outcome. While keeping it fair, we manipulate the association of the participant actions with the allocation of dictatorship in the dictator game to find differences in subsequent monetary transfers that are likely to be mediated by their subjective perceptions of the fairness of said association.

## Experimental design

The experiment builds upon the dictator game where we introduce a peculiar role allocation procedure as an additional stage that precedes it.

In the classic dictator game [14], there is a monetary endowment (essentially, ‘manna from heaven’) to be shared between two anonymous players. One of the players (the dictator) decides how much of the endowment to *transfer* to the other, passive player (the recipient) while keeping the rest for themselves. The roles are assigned randomly and the game is played only once, which makes it a popular means of studying the human nature of self-interest, perceptions of fairness and merit [15]. The aforementioned formulation of the game—i.e., with the random role allocation—constitutes our baseline treatment.

**Fig 1:**
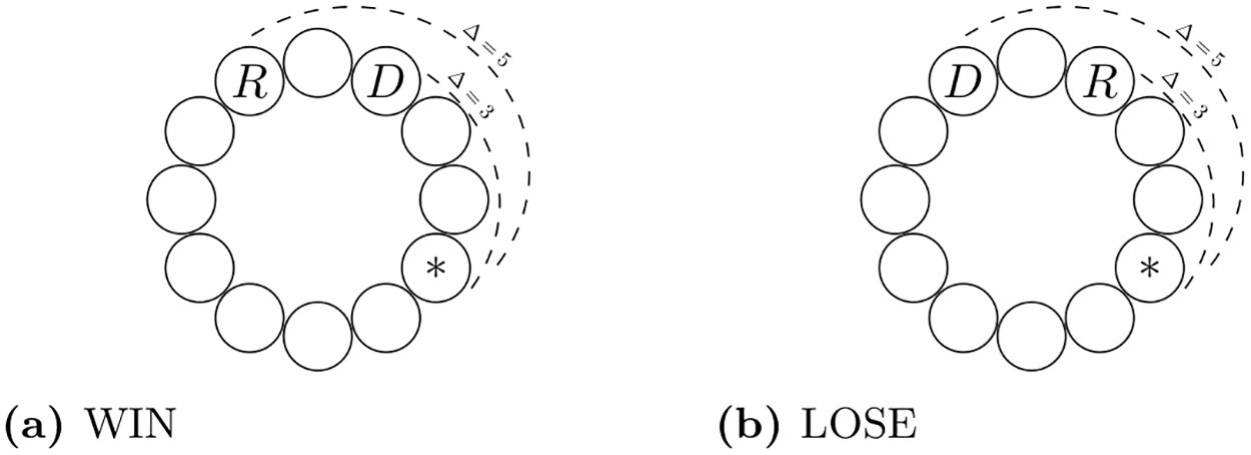
Role allocation procedure in treatments WIN and LOSE. Asterisk (*) denotes the bead randomly selected by the computer. Between the two participants, the *better* guess acquires the role of the dictator (D) in treatment WIN (a) and the role of the recipient (R) in treatment LOSE (b). The better guess is the one closer, in either direction, to the selected bead. There were 19 beads to choose from in the actual experiment.

In the other two treatment conditions, the participants are presented with what we refer to as a necklace consisting of 19 beads. We wanted to have a relatively large action space while at the same time, minimizing scope for focal points implied by visual symmetry, familiarity with a clock dial etc. (see Fig 1). One of the beads is randomly selected by the computer and both participants are asked to guess it. In treatment WIN, the better guess (i.e., the one closer, in either direction, to the selected bead) results in acquiring the dictator role whereas in treatment LOSE, the better guess results in acquiring the recipient role. An alternative way of thinking about one’s objective in LOSE is that one has to pick a bead *further away* from the selected one. In case of a tie in either treatment condition, the roles were to be assigned randomly. This happened only twice during the course of the experiment.

In our opinion, it is self-evident that the design allows for neither ability nor effort to be applied by either participant to affect their chance of being assigned dictatorship and therefore, leaves the question of its merit open to interpretation. At the same time, both treatment conditions remain fair in that both participants have equal chances of acquiring said power.

In each treatment condition, the dictator is given 50 indivisible experimental currency units (ECU) to distribute between themselves and the recipien. We used an exchange rate of 10 ECU to 1 EUR. We employ what is known as the strategy method [16] to elicit the transfer decisions from both participants in the role of the dictator and then use the role allocation procedure to determine the payoff outcomes for a given pair (if anything, this makes the design more conservative; see [17]).

From the participant perspective, the general order of events is as follows: (i) learn the rules of the dictator game and role allocation procedure; (ii) guess the bead selected by the computer (unless in the baseline condition); (iii) make the transfer decision in the role of the dictator; and (iv) find out the role allocations and the resulting payoff outcomes. Immediately after the transfer decision (i.e., before the roles and consequently, payoffs are revealed), the participants are asked to evaluate the role allocation procedure in terms of fairness and merit (see What drives selective heeding?). For details, see S1 File.

From our conjecture of selective heeding, it follows that the role allocation procedure is considered by the dictators when it is in their favor and disregarded otherwise. This leads to the following two hypotheses:

(i) average transfer in treatment WIN is lower than the baseline;
(ii) average transfer in treatment LOSE is not different from the baseline.

Findings in line with these hypotheses would support our conjecture that people tend to attribute merit to their irrelevant actions but not to irrelevant actions of others.

## Ethics statement

The experiment complied with the ethical regulations of the Chair of Empirical and Experimental Economics at the Friedrich Schiller University Jena. The participants were registered at the Economics Laboratory and accepted the general terms of participation in economic experiments.

## Results

The experiment was conducted with 130 participants at the Economics Laboratory of the Friedrich Schiller University Jena. It was programmed in z-Tree [18] and the recruitment was done with the help of ORSEE [19].

The participants were allocated to the treatment conditions randomly and interacted via computer terminals to preserve their anonymity. The game was played once and no repeat participation was allowed. All treatment conditions were run concurrently over 9 sessions. Each session concluded within 30 minutes and the average payment was 5.0 EUR (including a show-up fee of 2.5 EUR).

One participant was excluded from the analysis, which had no qualitative effect on the results. We suspect that they were not properly motivated by our incentive scheme since they transferred 80% of their endowment as the dictator. Among other things, they were 42 years old and were not a student. The final sample of 129 observations includes 81 females, 74 undergraduates and 32 majors in Business Administration or Economics. The average participant age is 24.6 years (SD 3.6) and the average laboratory experience is 7.6 experiments (SD 6.1).

**Fig 2:**
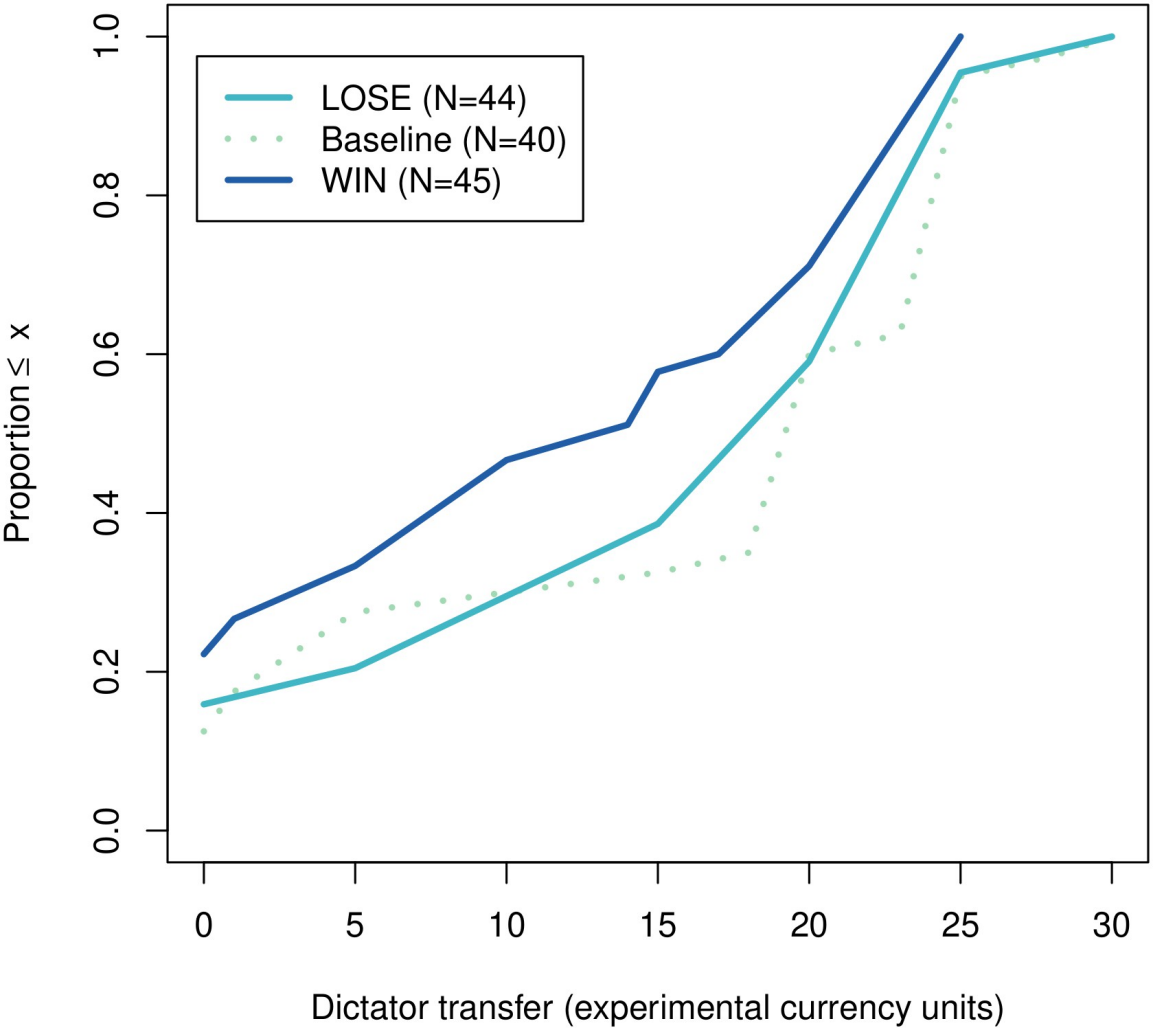
Empirical distribution function of the dictator transfer by treatment condition.

In total, we have 40, 44 and 45 observations in the baseline, LOSE and WIN conditions, respectively. The empirical distribution functions of the dictator transfers are presented in Fig 2.

As one can see, the empirical distribution function of the dictator transfer in the WIN condition is stochastically dominated by the other two whereas no such claim can be made as far as the comparison between the baseline and LOSE conditions. The resulting average transfers are equal to 16.9, 17.1 and 13.1 (ECU) in the baseline, LOSE and WIN conditions, respectively.

To test for the statistical significance of the observed differences between the treatment conditions, we estimate the specification given below using ordinary least squares and subsequently perform the t-test on the coefficient estimates at the respective treatment variables:

$$\begin{array}{c} y_{i}=\alpha +\beta \times WIN_{i}+\gamma \times LOSE_{i}+\epsilon_{i}, \end{array}$$ (1)

where *i* indexes the participant; *WIN*_*i*_ and *LOSE*_*i*_ equal one if the participant is assigned to the respective treatment condition and zero otherwise. A complementary specification adds a number of control variables capturing the effects of age (quadratic), gender, educational background (undergraduate, Business Administration or Economics major) and previous experience in laboratory experiments (quadratic).

The regression results are summarized in Table 1. At the significance level of 5%, we reject the null hypothesis that the average transfer in the WIN condition is not smaller than the baseline (one-tailed *p*-value of 0.031 or 0.039, with or without controls, according to our directional hypothesis (see Experimental design) and at the same time, cannot reject the null hypothesis that the average transfer in the LOSE condition is equal to the baseline (two-tailed *p*-value of 0.582 or 0.946, with or without controls).

**Table 1:** Regression results (s.e. in parentheses).

	Transfer	
Intercept	16.900 ***	-27.483
	(1.551)	(28.337)
WIN	-3.789 **	-3.943 **
	(2.132)	(2.094)
LOSE	0.145	-1.175
	(2.143)	(2.132)
Controls	–	+
R-squared (N = 129)	0.035	0.140
Cohen’s *d*: WIN	0.382	
Cohen’s *d*: LOSE	0.015	

Dictator transfer measured in experimental currency units, perceptions of fairness and merit measured on a 1–7 scale. Also, estimated effect sizes relative to the baseline.

Controls: age (quadratic), gender, undergraduate, Business Administration or Economics major, laboratory experience (quadratic).

Significance (one-tailed for WIN):

*** ≡ *p* < 0.01;

** ≡*p* < 0.05;

* ≡ *p* < 0.1.

As additional evidence, consider the effect sizes of the WIN and LOSE manipulations. Using Cohen’s *d* as a quantitative measure results in the actual estimates of 0.382 and 0.015, which we interpret as practically important and practically unimportant, respectively.

In summary, we find empirical support for both of our research hypotheses—i.e., the participants exhibit *selective heeding* as far as giving credit to one’s action. If dictatorship is assigned through an arbitrary procedure that favors them, they tend to make lower transfers than those who are assigned dictatorship in a completely random fashion. However, if an arbitrary procedure favors the other player instead, the dictator transfers do not reflect that.

## What drives selective heeding?

In anticipation of the above findings, we measured the participants’ perceptions of the fairness of the role allocation procedure (hereafter, *fairness*) as well as their perceptions of how much the selected person deserved to determine the payoff outcomes relative to the other, not selected person (hereafter, *merit*).

The perception of fairness was measured on a 1–7 scale where 1 implies an absolutely unfair procedure and 7 implies an absolutely fair procedure as far as the chances of both participants to determine the payoff outcomes. Translated from German the question reads as follows: “As far as the chances of both participants to determine the actual transfer, is the procedure fair?” 〈Disagree completely ∼ Agree completely〉.

The perception of merit was measured on a 1–7 scale where 7 implies that the selected player deserves to determine the payoff outcomes more than the other player, 1 implies the opposite and 4 implies that the two deserve it equally. Translated from German the question reads as follows: “Do you think that the selected person deserves to determine the actual transfer as much as the not-selected person?” 〈The selected person deserves it more ∼ Both deserve it equally ∼ The not-selected person deserves it more〉.

Our conjecture was that those perceptions could be illuminating in explaining the differences in the transfers should there be any. As far as the identification strategy, we can think of two major, mutually exclusive, approaches.

One is to view the subjective perceptions as post hoc rationalizations that the participants may use to explain their transfer decisions after the fact [20] (if anything, to themselves). From this perspective, our manipulations can have two effects: one on the transfer amounts and the other on the elicited perceptions. The former, however, has temporal precedence over the latter and so the perceptions cannot be causally responsible for the transfers.

Alternatively, one can assume the effect of our manipulations to be fully internalized by the perceptions, which then in turn can cause changes in the transfer amounts. From this perspective, there is a chain reaction from the manipulation to the perception to the transfer.

Statistically speaking, the first approach is to treat the perceptions of fairness and merit as independent variables in a model that is otherwise identical to the one represented by Eq (1). The second approach is to replace the treatment variables with the perception metrics in said equation.

**Fig 3:**
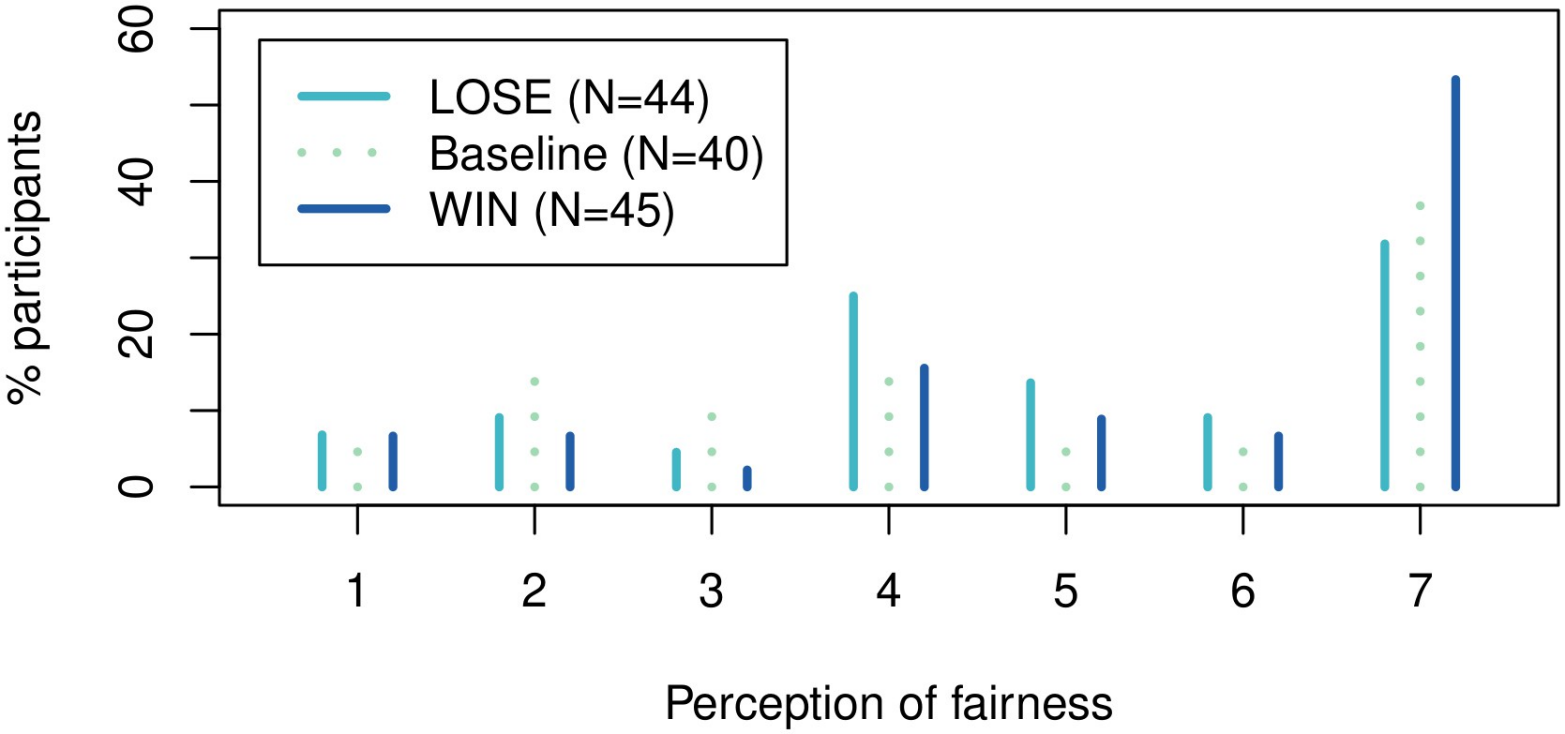
Perception of the fairness of the role allocation procedure by treatment condition. 1 implies an absolutely unfair procedure while 7 implies an absolutely fair procedure as far as the chances of both participants to determine the payoff outcomes.

**Fig 4:**
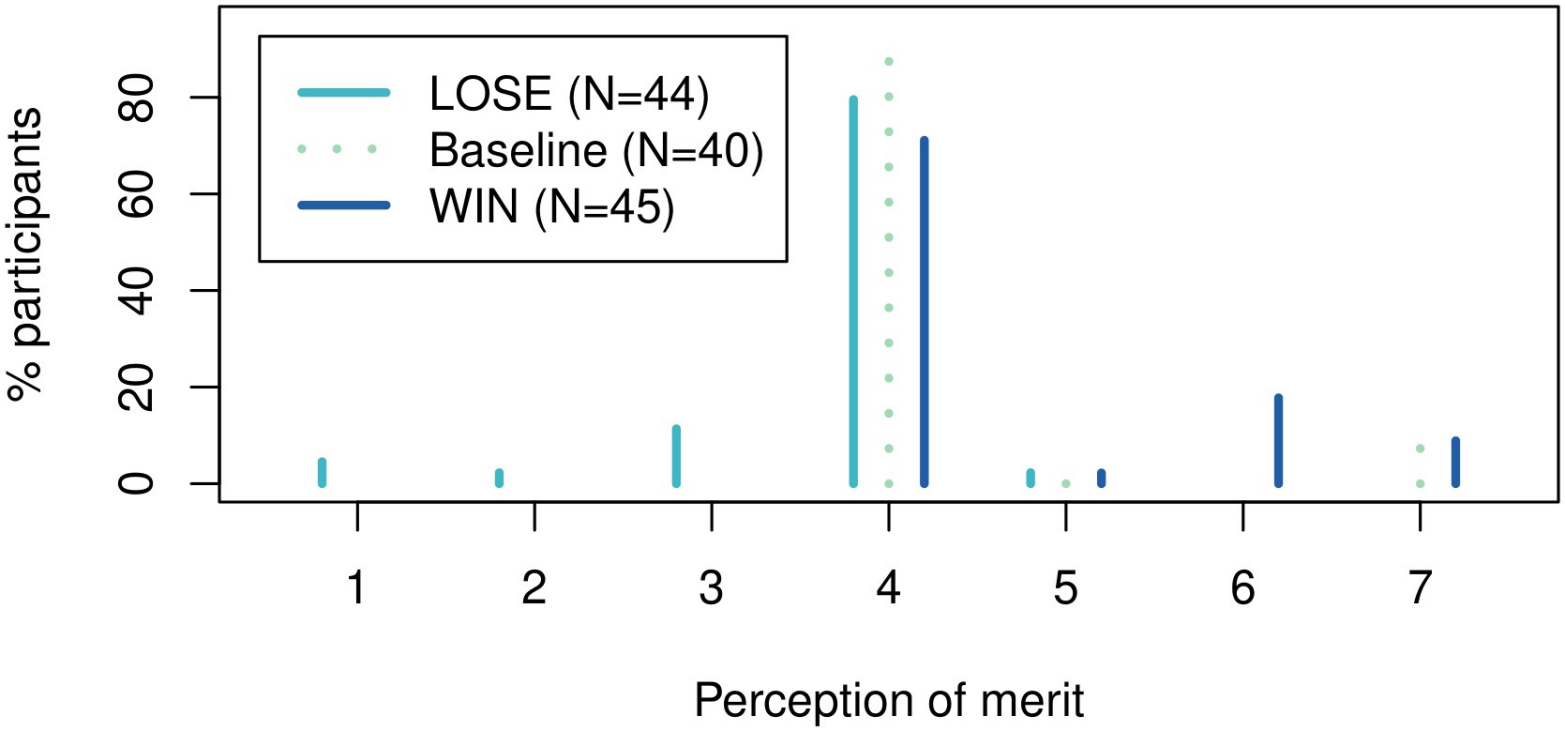
Perception of the relative merits of the participants by treatment condition. 7 implies that the selected player deserves to determine the payoff outcomes more than the other, 1 implies the opposite and 4 implies that the two deserve it equally.

We present the results following the former (our preferred) approach first. The empirical distributions of the participant perceptions are presented in Figs 3 and 4 and the regression results are summarized in Table 2.

**Table 2:** Regression results (s.e. in parentheses).

	Fairness		Merit	
Intercept	4.650 ***	-1.393	4.275 ***	0.499
	(0.324)	(6.118)	(0.142)	(2.666)
WIN	0.817 **	0.818 **	0.369 **	0.358 **
	(0.445)	(0.452)	(0.195)	(0.197)
LOSE	0.191	0.046	-0.548 ***	-0.568 ***
	(0.447)	(0.460)	(0.196)	(0.201)
Controls	–	+	–	+
R-squared (N = 129)	0.029	0.074	0.158	0.202
Cohen’s *d*: WIN	0.389		0.385	
Cohen’s *d*: LOSE	0.092		0.697	

Perceptions of fairness and merit measured on a 1–7 scale. Also, estimated effect sizes relative to the baseline.

Controls: age (quadratic), gender, undergraduate, Business Administration or Economics major, laboratory experience (quadratic).

Significance (one-tailed for WIN):

*** ≡ *p* < 0.01;

** ≡*p* < 0.05;

* ≡ *p* < 0.1.

As far as the fairness of the role allocation procedure, the average perception is equal to 4.65, 4.84 and 5.47 in the baseline, LOSE and WIN conditions, respectively. At the significance level of 5%, the participants in the LOSE condition do not perceive the role allocation procedure to be any different from the baseline (two-tailed *p*-value of 0.920 or 0.670, with or without controls). In the WIN condition, however, the role allocation procedure is perceived to be significantly fairer (one-tailed *p*-value of 0.037 or 0.034, with or without controls), which is in line with our directional hypothesis as presented in Experimental design.

These findings are in line with the effect our manipulations have on the actual monetary transfers, which suggests that selective heeding may be driven by one’s perception of fairness. The Cohen’s *d* estimates of 0.092 and 0.389, which we interpret as practically unimportant and practically important, respectively, provide further support.

As far as the relative merits of the participants, the average perception is equal to 4.28, 3.73 and 4.64 in the baseline, LOSE and WIN conditions, respectively. At the significance level of 5%, the participants believe that the selected player deserves to determine the payoff outcomes significantly less in the LOSE condition (two-tailed *p*-value of 0.005 or 0.006, with or without controls) and more so in the WIN condition (one-tailed *p*-value of 0.036 or 0.030, with or without controls, in line with our directional hypothesis as presented in Experimental design. With the respective Cohen’s *d* estimates of 0.697 and 0.385, both effects are also practically important.

Finding that the participants perceive the selected player to be less deserving of dictatorship in the LOSE condition is particularly interesting. Also, note that the value of 3.73 implies that in fact, the not-selected player deserves dictatorship more than the selected one. This suggests that the average transfer should not only be larger than the baseline but actually exceed 50% of the total endowment.

We thus conclude that, from the post hoc rationalization perspective, the observed differences in the dictator transfers (i.e., selective heeding) have more to do with changes in the participant perceptions of the fairness of the role allocation procedure rather than with changes in their perceptions of the relative merits of the players.

Table 3 presents the regression results from the alternative perspective, according to which the perceptions of fairness and merit can have direct causal effects on the transfer amount. In light of that, one would expect self-centered dictators to give less the more they believe the allocation procedure to be fair as well as the more they believe to deserve their role.

**Table 3:** Regression results (s.e. in parentheses).

	Transfer	
Intercept	21.104 ***	-26.419
	(4.600)	(28.419)
Fairness	-0.282	-0.505
	(0.427)	(0.420)
Merit	-0.964	-1.209 *
	(0.909)	(0.898)
Controls	–	+
R-squared (N = 129)	0.012	0.134

Dictator transfer measured in experimental currency units, perceptions of fairness and merit measured on a 1–7 scale.

Controls: age (quadratic), gender, undergraduate, Business Administration or Economics major, laboratory experience (quadratic).

Significance (one-tailed):

*** ≡ *p* < 0.01;

** ≡*p* < 0.05;

* ≡ *p* < 0.1.

As one can see, the coefficient estimates at Fairness and Merit are both negative, which is in line with said expectations. These results are not statistically significant at the 5% level, though (one-tailed *p*-value of 0.255 or 0.116, with or without controls, for the perception of fairness and 0.145 or 0.090 for the perception of merit).

## Discussion

We examine a modified version of the dictator game where dictatorship is assigned by a fair procedure that is linked to the participant actions but that is in effect completely random. This enables the dictator to entertain arbitrary beliefs as far as the relative entitlements of the players to the endowment. Subtle modifications to the procedure illustrate that these beliefs are not only dependent on the outcome but are also inherently self-serving in that the participants tend to give credit to themselves if the result of a random draw is in their favor but do not give credit to others if it is not.

We refer to this asymmetry as selective heeding and investigate two potential explanatory factors behind it such as the perceptions of fairness and merit. It appears that the observed differences in the dictator transfers are consistent with the changes in the perception of the fairness of the role allocation procedure but are not consistent with the changes in the perception of the relative merits of the participants as induced by our manipulations.

Our findings add to the literature on procedural preferences [21–26] as well as to the more general research on self-centered behavior and motivated reasoning [27–29]. As long as people can find situational excuses they tend to relax their moral standards and consequently, behave more selfishly. Existing literature presents numerous examples in various contexts: individual ability [30], personal culpability [31], real effort [32]. We show that ‘winning’ a game of pure chance is as good of a reason as one could possibly have. Moreover, there seems to be some inherent asymmetry to the logic: I win it’s fair, you win it’s not.

The self-centered phenomenon that we document here goes beyond the notions of ‘self-serving bias’ [33, 34] or ‘attribution bias’ [35, 36] that refer to commonly observed tendencies to overestimate one’s personal input in case of success and to underestimate it in case of failure. Our participants state that they do not deserve dictatorship all the time. It just so happens that they are not willing to put their money where their mouth is.

## Supporting information

S1 File Participant instructions. (PDF)

S1 Data (CSV)

S2 Data (TXT)
