# Supplementary material for: I win it’s fair, you win it’s not. Selective heeding of merit in ambiguous settings
Source: PLoS One. 2023 Jan 6;18(1):e0279865. doi: 10.1371/journal.pone.0279865 (PMC9821486; doi:10.1371/journal.pone.0279865)
Supplement: S1 File — (PDF) [file pone.0279865.s001.pdf]

S1 Participant instructions

{*All treatments*}

Experiment Instructions

Welcome and thank you for participating in this experiment. Please switch off your mobile phones and remain silent. If you have any questions, please raise your hand and experimenter will answer you privately.

For your participation you will receive a show-up fee of 2.50 EUR. During the experiment you can earn additional money. Your additional earnings depend on your own decisions, decisions of other participants as well as on chance. During the whole experiment your anonymity is guaranteed. Your additional earnings will be expressed in ECU (experimental currency unit) that will be converted into EUR at the end of the experiment using the following exchange rate:

$$1 \text{ ECU} = 0.10 \text{ EUR}.$$

Structure of the Experiment

At the beginning of the experiment all participants will be matched into pairs. You stay within the same pair throughout the experiment.

{*Baseline only*}

The experiment will continue as follows.

The participants in the pair will be randomly assigned one of the two roles: **Participant A** or **Participant B**. Each role is equally likely to be selected.

{*Treatments WIN and LOSE only*}

The experiment consists of two parts.

In the first part of the experiment, you and the other person in your pair will be presented with a 'necklace' consisting of several beads. One of these beads will be randomly selected by the computer.

Each of you will be asked to guess which bead it was. We will then compare your guesses and assign the role of **Participant A** to the person whose guess was closer to the bead selected by the computer (in any direction). The other person will be assigned the role of **Participant B**[1]

Consider the following examples, where the computer randomly selects the bead labeled as $*$, one person selects the bead labeled as X, and the other person selects the bead labeled as Y:

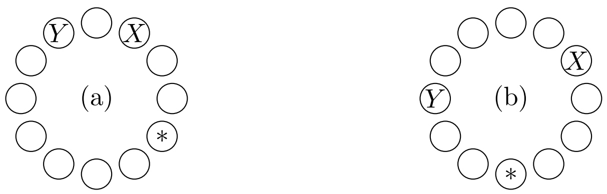

The roles will then be assigned in the following way:

(a) Person X will be assigned the role of Participant A, and person Y will be assigned the role of Participant B.

[1] In case of a tie, the roles will be assigned randomly (each role is then equally likely to be selected).

(b) Person X will be assigned the role of Participant B, and person Y will be assigned the role of Participant A.

{ *All treatments;*
‘I’ = ‘A’ in the baseline and WIN, and ‘B’ in LOSE;
‘J’ = ‘B’ in the baseline and WIN, and ‘A’ in LOSE; }

[In the second part of the experiment,]{*WIN & LOSE*} **Participant I** receives an endowment of 50 ECUs and decides how much from this amount he would like to send to Participant J. Participant I can specify any amount between 0 and 50 ECU in steps of 1 ECU.

The amount specified by Participant I will be deducted from this endowment and transferred to Participant J, and the rest will remain for Participant I.

Participant J does not make any decisions in this [situation{*Baseline*}; part of the experiment{*WIN & LOSE*}].

Before you know the result of assigning the roles, you will be asked to decide as Participant I. After you and the other person in your pair have made your decisions in the role of Participant I, the computer will [randomly select one of you to be the actual Participant I{*Baseline*}; assign the roles according to your guesses in the first part of the experiment{*WIN & LOSE*}] and then implement the respective decision for your pair.

At the end of [this decision situation{*Baseline*}; the second part{*WIN & LOSE*}], you will be informed about the following: the role you have been assigned to, the transfer implemented for your pair, as well as your final payment for participating in the experiment.

We will conclude the experiment by asking you to fill out a short questionnaire.

If you have read these instructions carefully and do not have any further questions, please click the ’Ready’ button on your screen. We will then test your understanding of the procedure with a few basic questions. As soon as everybody has answered those questions correctly, the experiment will begin.

Good luck!
